# Supplementary material for: Differential regulation of breast cancer bone metastasis by PARP1 and PARP2
Source: Nat Commun. 2020 Mar 27;11:1578. doi: 10.1038/s41467-020-15429-z (PMC7101362; doi:10.1038/s41467-020-15429-z)
Supplement: Supplementary file 1 — Supplementary Information [file 41467_2020_15429_MOESM1_ESM.pdf]

*Supplementary Information*

# Differential Regulation of Breast Cancer Bone Metastasis by PARP1 and PARP2

Zuo et al.

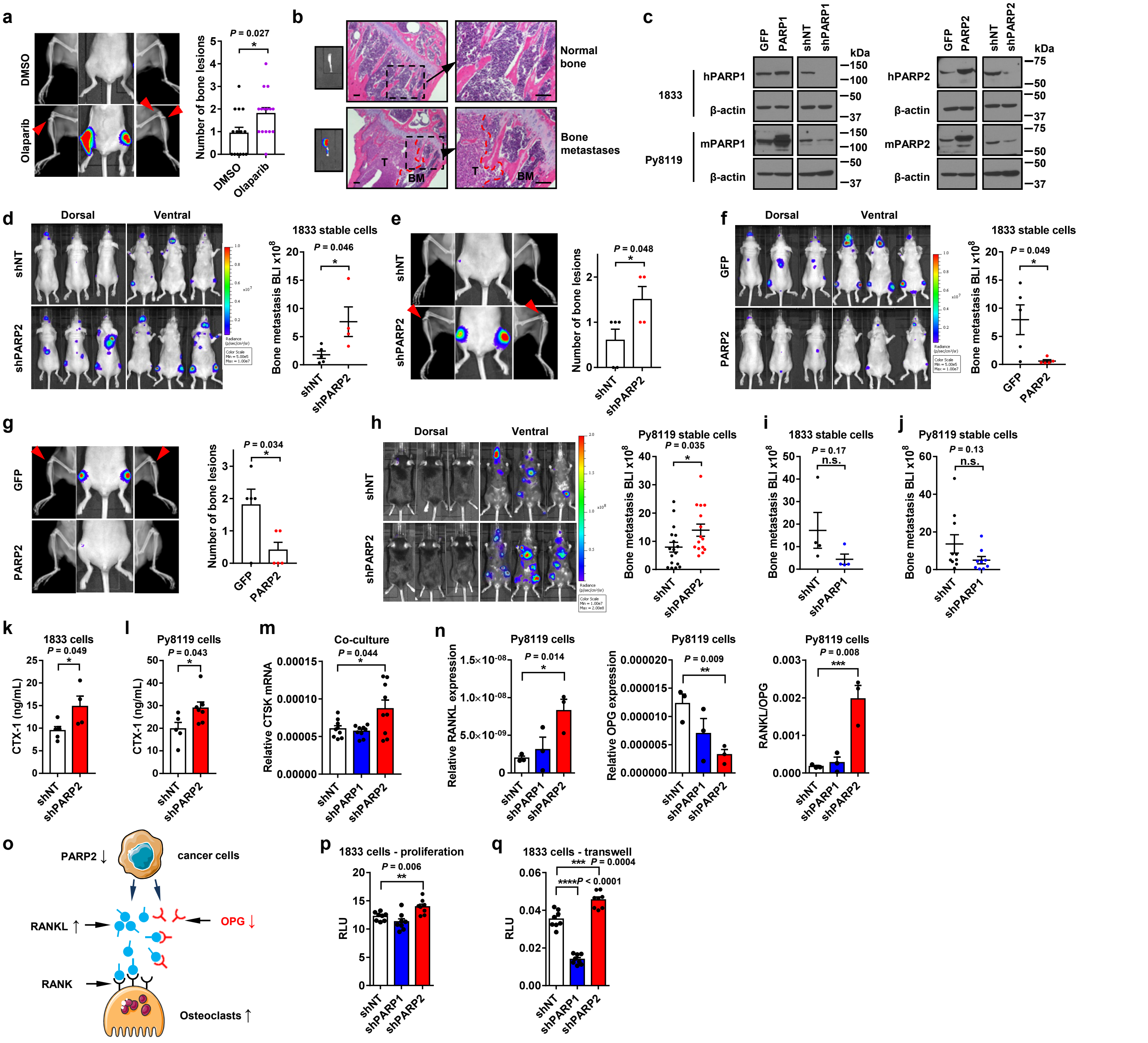


**Supplementary Fig. 1 | PARP2 deficiency in cancer cells increases breast cancer bone metastasis. a**, Representative X-ray images and bone lesion quantification of nude mice intracardiacally injected with 1833 cells and treated with DMSO or olaparib ($n$ = 14). **b**, H&E staining of the distal femur with or without bone metastases. Staining was repeated twice with different mice and showed similar results. Scale bar, 0.1 mm. **c**, Western blot analyses showing the efficiency of stable overexpression and knocking down of PARP1 or PARP2 in 1833 cells and Py8119 cells. Western blot analyses were repeated twice and showed similar results. **d**, **e**, Nude mice injected with PARP2KD 1833 cells ($n$ = 4) developed more bone metastases (**d**) and bone lesions (**e**) than control 1833 cells ($n$ = 5). **f**, **g**, Bone metastasis (**f**) and bone lesions (**g**) of PARP2-overexpressing 1833 cells in nude mice ($n$ = 5) were reduced compared with control cells ($n$ = 4). **h**, B6 mice injected with PARP2KD Py8119 cells developed more bone metastases than control Py8119 cells ($n$ = 15). **i**, **j**, Nude mice injected with PARP1KD 1833 cells (**i**, $n$ = 5) or B6 mice injected with PARP1KD Py8119 cells (**j**, $n$ = 9) had similar bone metastases as respective control 1833 cells ($n$ = 4) or Py8119 cells ($n$ = 10). **k**, **l**, Serum CTX-1 levels in nude mice (**k**) or B6 mice (**l**) injected with PARP2KD 1833 cells ($n$ = 4) or Py8119 cells ($n$ = 7) were higher than mice injected with control cells. **m**, Osteoclast precursors had increased expression of osteoclast marker gene CTSK after co-culturing with Py8119-shPARP2 cells for 3 days as compared to Py8119-shNT or shPARP1 cells ($n$ = 9). **n**, Py8119-shPARP2 cells had increased RANKL mRNA (left), decreased OPG mRNA (middle) and elevated RANKL/OPG ratio (right) as compared to Py8119-shNT or shPARP1 cells ($n$ = 3). **o**, A diagram showing that PARP2KD in breast cancer cells increases osteoclast differentiation. **p**, PARP2 levels negatively correlated with the proliferation rate of 1833 cells ($n$ = 8). **q**, Migration of PARP2KD 1833 cells through transwell was increased ($n$ = 8). Data represent mean ± SEM, *$P$ < 0.05, **$P$ < 0.01, ***$P$ < 0.001, ****$P$ < 0.0001; n.s., non-significant. Two-sided Student's $t$-test was used to calculate statistical difference. Source data are provided as a Source Data file.

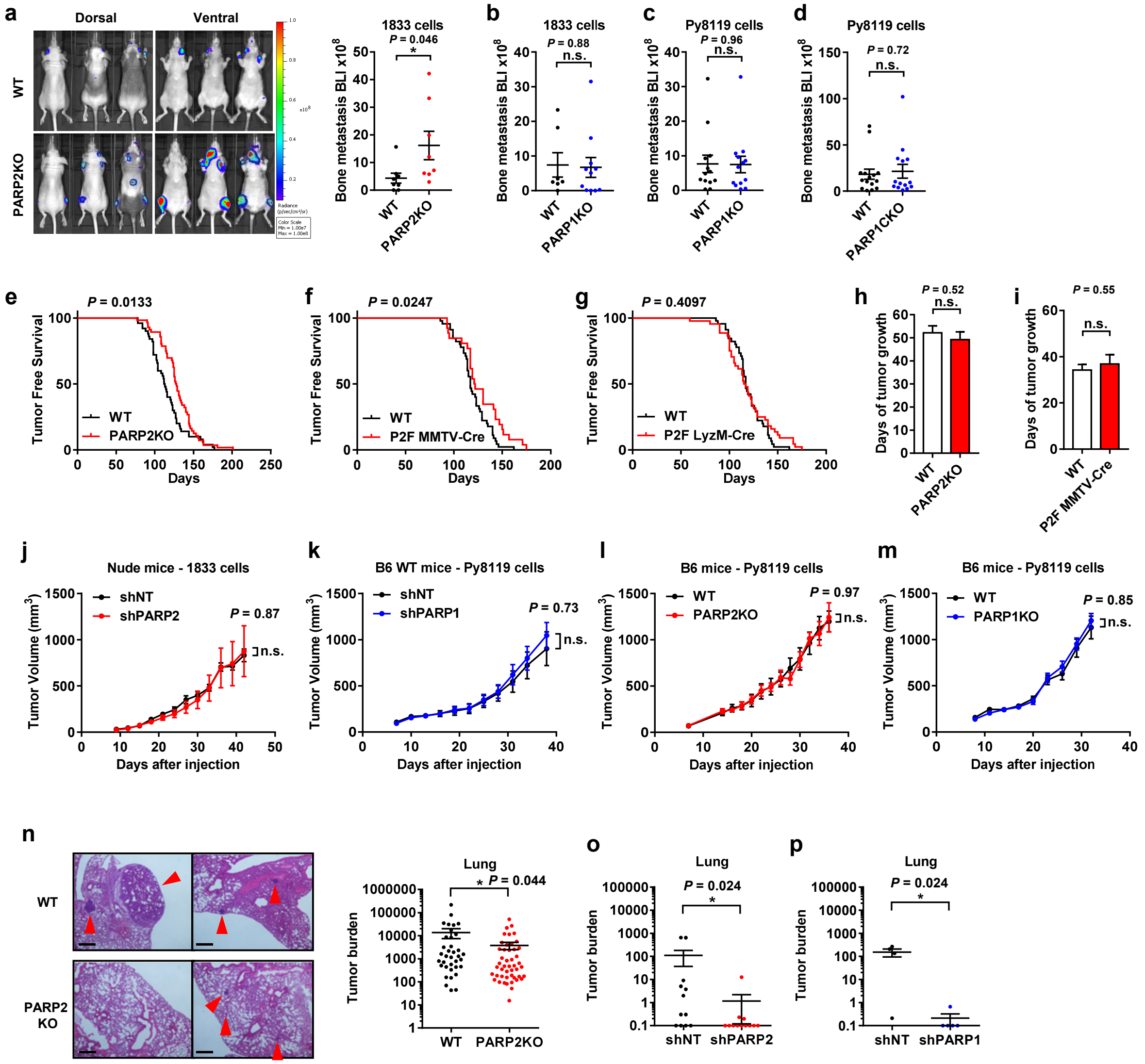


**Supplementary Fig. 2 | PARP2 deficiency does not affect primary tumor growth but suppresses lung metastasis.** **a**, Bone metastasis of 1833 cells was increased in PARP2KO nude mice compared with WT nude mice (*n* = 8). **b**, Bone metastasis of 1833 cells was similar in nude WT (*n* = 7) or PARP1KO mice (*n* = 11). **c**, Bone metastasis of Py8119 cells was similar in B6 WT or PARP1KO mice (*n* = 13). **d**, Bone metastasis of Py8119 cells was similar in B6 WT or PARP1CKO (*n* = 14) mice. **e-g**, PARP2KO (**e**, *n* = 56) and PARP2 flox/flox (P2F) + MMTV-Cre (**f**, *n* = 26), but not P2F + lyzM-Cre (**g**, *n* = 44), increased tumor free survival of MMTV-PyMT mice compared with respective control PARP2WT (**e**, *n* = 50) or P2F (**f**, **g**, *n* = 45) mice. Two-sided log-rank test was used to calculate statistical difference. **h**, **i**, The time of primary tumor growth between tumor onset and end point (maximal volume limit) of mice in **e** and **f** was not altered by PARP2 deletion. **j-m**, Primary mammary tumor growth were similar for PARP2KD 1833 cells vs. control 1833 cells in nude mice (**j**, *n* = 5), PARP1KD Py8119 cells vs control Py8119 cells in B6 WT mice (**k**, *n* = 13), Py8119 cells in WT vs. PARP2KO B6 mice (**l**, *n* = 7), and Py8119 cells in WT vs. PARP1KO mice (**m**, *n* = 10). Statistical analysis was performed using two-way ANOVA with Sidak's multiple comparison. **n**, Left, H&E staining of spontaneous lung metastases in PARP2KO MMTV-PyMT mice (scale bar, 25 μm). Arrows, lung metastases. Right, PARP2KO MMTV-PyMT mice had reduced tumor burden of spontaneous lung metastases compared with WT MMTV-PyMT mice (*n* = 36). **o**, **p**, Tumor burden of spontaneous lung metastases was less in B6 mice injected with PARP2KD (**o**, *n* = 12) or PARP1KD (**p**, *n* = 5) Py8119 cells at mammary fat pad than mice with control cells. Data represent mean ± SEM, *$P$ < 0.05; n.s., non-significant. **a-d**, **h**, **i**, Two-sided Student's *t*-test was used to calculate statistical difference. **n-p**, Two-sided Mann-Whitney *U* test was performed (non-normally distributed data). Source data are provided as a Source Data file.

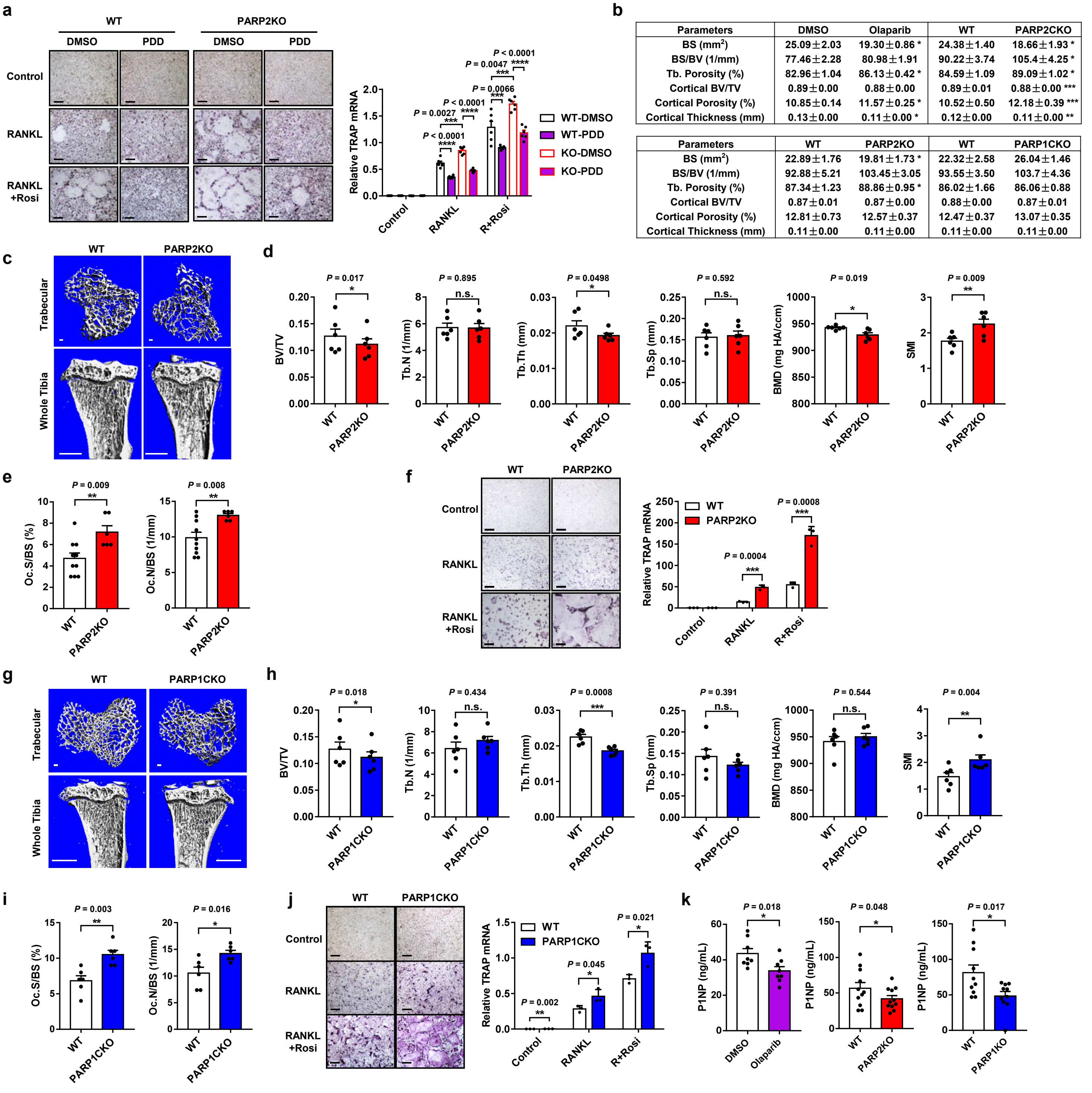


b

| Parameters | DMSO | Olaparib | WT | PARP2CKO |
|---|---|---|---|---|
| BS ($mm^2$) | 25.09±2.03 | 19.30±0.86 * | 24.38±1.40 | 18.66±1.93 * |
| BS/BV (1/mm) | 77.46±2.28 | 80.98±1.91 | 90.22±3.74 | 105.4±4.25 * |
| Tb. Porosity (%) | 82.96±1.04 | 86.13±0.42 * | 84.59±1.09 | 89.09±1.02 * |
| Cortical BV/TV | 0.89±0.00 | 0.88±0.00 | 0.89±0.01 | 0.88±0.00 *** |
| Cortical Porosity (%) | 10.85±0.14 | 11.57±0.25 * | 10.52±0.50 | 12.18±0.39 *** |
| Cortical Thickness (mm) | 0.13±0.00 | 0.11±0.00 * | 0.12±0.00 | 0.11±0.00 ** |

| Parameters | WT | PARP2KO | WT | PARP1CKO |
|---|---|---|---|---|
| BS ($mm^2$) | 22.89±1.76 | 19.81±1.73 * | 22.32±2.58 | 26.04±1.46 |
| BS/BV (1/mm) | 92.88±5.21 | 103.45±3.05 | 93.55±3.50 | 103.7±4.36 |
| Tb. Porosity (%) | 87.34±1.23 | 88.86±0.95 * | 86.02±1.66 | 86.06±0.88 |
| Cortical BV/TV | 0.87±0.01 | 0.87±0.00 | 0.88±0.00 | 0.87±0.01 |
| Cortical Porosity (%) | 12.81±0.73 | 12.57±0.37 | 12.47±0.37 | 13.07±0.35 |
| Cortical Thickness (mm) | 0.11±0.00 | 0.11±0.00 | 0.11±0.00 | 0.11±0.00 |

**Supplementary Fig. 3 | PARP2KO and PARP1CKO increase osteoclast differentiation. a**, Treatment with 1 μM PARG inhibitor, PDD 00017273 (PDD), suppressed *in vitro* osteoclast differentiation of WT or PARP2KO cultures ($n$ = 3). Left, TRAP staining (scale bar, 25 μm); right, TRAP expression. **b**, Other trabecular and cortical bone parameters in μCT. BS, bone surface. **c**, **d**, μCT of the tibiae from PARP2KO mice showed reduced BV, Tb.Th, BMD and increased SMI compared with littermate WT control mice (2-month-old, male, $n$ = 6). **c**, Images of the trabecular bone of the tibial metaphysis (top) (scale bar, 100 μm) and the entire proximal tibia (bottom) (scale bar, 1 mm) from WT or PARP2KO mice. **d**, Trabecular bone parameters. **e**, Histomorphometry of TRAP-stained bone sections from the distal femur showed that PARP2KO mice had more osteoclasts than WT mice (2-month-old, male, $n$ = 6). **f**, PARP2KO cultures showed increased osteoclast differentiation ($n$ = 3). Left, TRAP staining (scale bar, 25 μm); right, TRAP expression. **g**, Images of the trabecular bone of the tibial metaphysis (top) (scale bar, 100 μm) and the entire proximal tibia (bottom) (scale bar, 1 mm) from WT or PARP1CKO mice. **h**, μCT of the tibiae showed that PARP1CKO mice had decreased BV, Tb.Th and increased SMI compared with WT littermates (2-month-old, male, $n$ = 6). **i**, Histomorphometry of the distal femur showed more osteoclasts in PARP1CKO mice than WT littermate controls (2-month-old, male, $n$ = 6). **j**, PARP1CKO cultures showed increased osteoclast differentiation ($n$ = 3). Left, TRAP staining (scale bar, 25 μm); right, TRAP expression. **k**, Serum bone formation marker P1NP levels of olaparib treated ($n$ = 8), PARP2KO ($n$ = 11) or PARP1KO ($n$ = 10) mice were lower. Data represent mean ± SEM, *$P$ < 0.05, **$P$ < 0.01, ***$P$ < 0.001, ****$P$ < 0.0001; n.s., non-significant. Two-sided Student's $t$-test was used to calculate statistical difference. Source data are provided as a Source Data file.

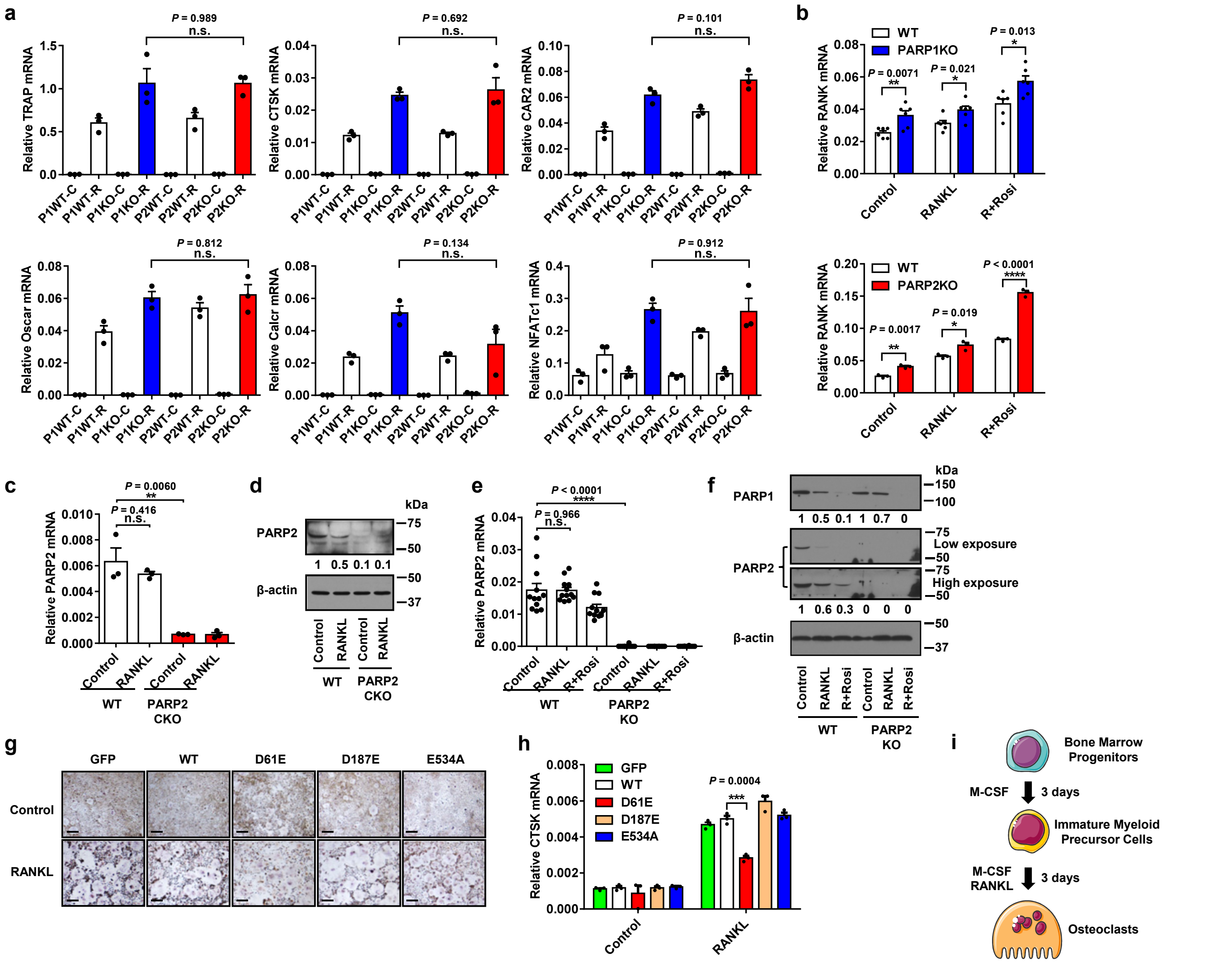

**Supplementary Fig. 4 | PARP1 and PARP2 regulate osteoclast similarly and are both cleaved during osteoclast differentiation. a**, **b**, The expression of osteoclast marker genes (**a**, *n* = 3) and RANK gene (**b**, *n* = 6 for PARP1KO, *n* = 3 for PARP2KO) in osteoclast differentiation assay was similarly up-regulated by PARP1KO or PARP2KO. P1, PARP1; P2, PARP2; C, control; R, RANKL; TRAP, tartrate-resistant acid phosphatase; CTSK, cathepsin K; CAR2, carbonic anhydrase 2; Oscar, osteoclast-associated receptor; Calcr, calcitonin receptor; NFATc1, Nuclear factor of activated T-cells, cytoplasmic 1; Rosi, rosiglitazone. **c**, Cultured PARP2CKO bone marrow cells had ~85% PARP2 deletion and RANKL did not alter PARP2 mRNA levels (*n* = 3). **d**, PARP2 protein level was reduced by RANKL in cultured WT bone marrow cells. Western blot analyses were repeated twice and showed similar results. **e**, **f**, PARP2KO bone marrow cells had complete PARP2 deletion. RANKL did not alter PARP2 mRNA (**e**, *n* = 12) but reduced PARP2 protein levels (**f**). Western blot analyses were repeated twice and showed similar results. **g**, **h**, Osteoclast differentiation assay with overexpression of control GFP, PARP2 WT, uncleavable mutants D61E or D187E, or enzymatic activity dead mutant E534A in RAW 264.7 murine macrophage cells. Only D61E mutant suppressed osteoclast differentiation. **g**, TRAP staining was repeated three times and showed similar results. **h**, CTSK expression (*n* = 3). **i**, A diagram showing osteoclast differentiation. Data represent mean ± SEM, **P* < 0.05, ***P* < 0.01, ****P* < 0.001, *****P* < 0.0001; n.s., non-significant. Two-sided Student's *t*-test was used to calculate statistical difference. Source data are provided as a Source Data file.

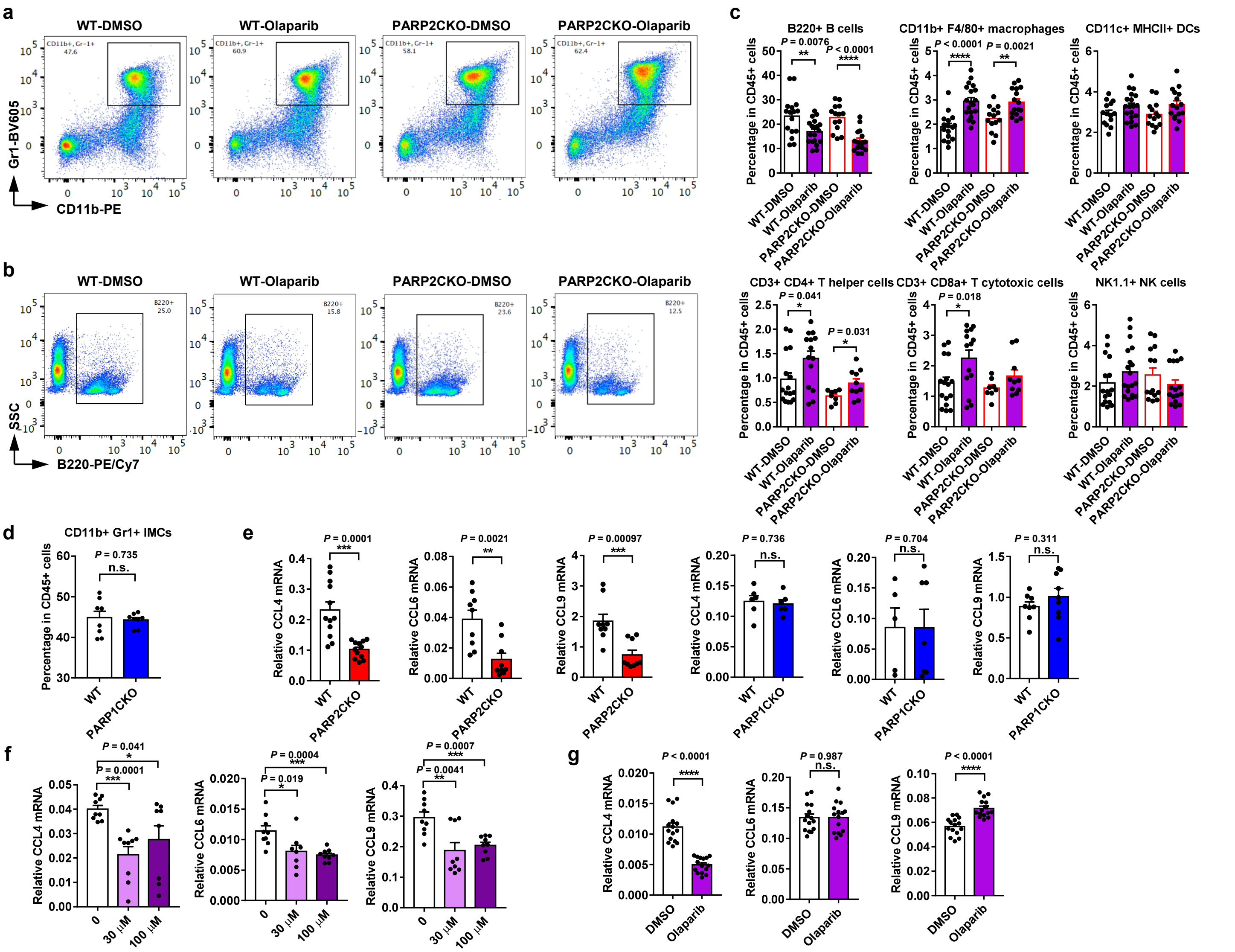


**Supplementary Fig. 5 | Additional analyses of bone marrow population and chemokine expression regulated by PARP. a, b**, Representative images of FACS analyses of CD11b+ Gr1+ IMC cells (**a**) and B220+ B cells (**b**) in bone marrow. **c**, Olaparib and PARP2CKO did not show similar effects on B220+ B cells, CD11b+ F4/80+ macrophages, CD11c+ MHCII+ DCs (dendritic cells), CD3+ CD4+ Th (T helper) cells, CD3+ CD8+ Tc (T cytotoxic) cells and NK1.1+ NK cells (natural killer cells) in FACS analyses of bone marrow cells from WT or PARP2CKO mice treated with DMSO or olaparib after injection with Py8119 cells ($n$ = 16). **d**, PARP1CKO did not alter IMCs in FACS analyses of bone marrow cells from WT or PARP1CKO mice intracardiacally injected with Py8119 cells ($n$ = 8). **e**, RT-qPCR showed that mRNA levels of CCL4, CCL6 and CCL9 were reduced in PARP2CKO IMCs ($n$ = 9) but not in PARP1CKO IMCs ($n$ = 6). **f**, The expression of CCL4, CCL6 and CCL9 mRNA in IMCs was suppressed by the treatment of 30 μM or 100 μM olaparib ($n$ = 9). **g**, CCL4 mRNA, but not CCL6 and CCL9 mRNA, was reduced in bone marrow cells of olaparib-treated B6 mice compared with DMSO-treated mice (2-month-old, female, 5-week daily treatment, $n$ = 5). Data represent mean ± SEM, *$P$ < 0.05, **$P$ < 0.01, ***$P$ < 0.001, ****$P$ < 0.0001; n.s., non-significant. Two-sided Student's $t$-test was used to calculate statistical difference. Source data are provided as a Source Data file.

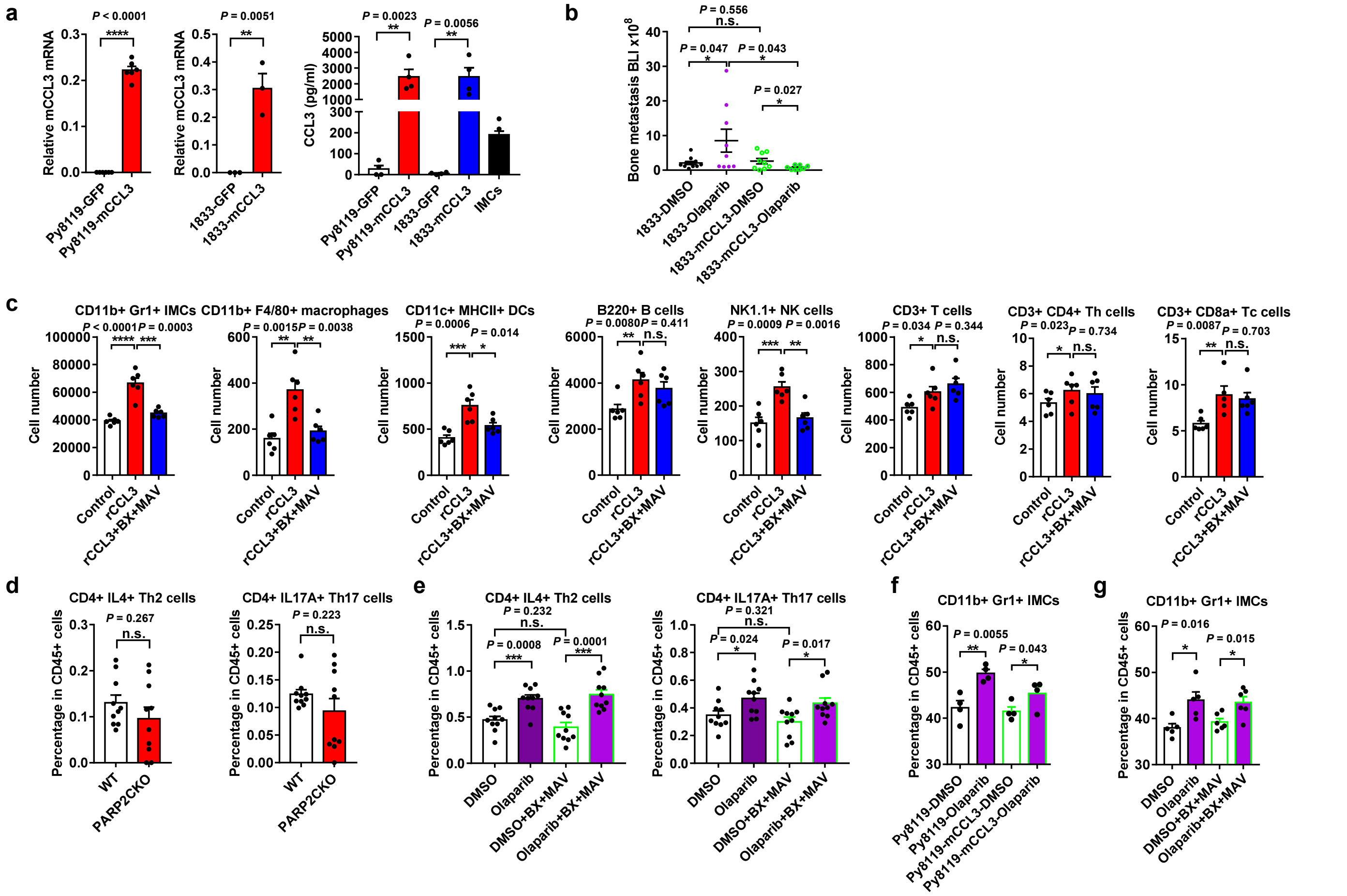


**Supplementary Fig. 6 | Effects of CCL3 on immune cells in bone marrow. a**, RT-qPCR and ELISA analyses showing the efficiency of stable overexpression of mCCL3 in Py8119 cells (*n* = 6) and 1833 cells (*n* = 3). CCL3 ELISA analyses were performed on conditioned medium cultured for 24 hours (*n* = 4). **b**, 1833-mCCL3 cells caused similar bone metastasis as 1833 cells after intracardiacally injection into nude mice, and olaparib increased bone metastasis of 1833 cells but decreased bone metastasis of 1833-mCCL3 cells (*n* = 10). **c**, 200 ng/mL recombinant murine CCL3 (rCCL3) triggered transwell migration of bone marrow IMCs, macrophages, DCs, NK, B and T cells. BX-471 and Maraviroc (BX and MAV, CCR1/5 antagonists, 1 μM) suppressed the CCL3-induced migration of IMCs, macrophages, DCs and NK cells (*n* = 6). **d**, PARP2CKO did not affect CD4+ IL4+ type 2 T helper cells (Th2) or CD4+ IL17A+ T-helper 17 cells (Th17) in FACS analyses of bone marrow cells from WT or PARP2CKO mice intracardiacally injected with Py8119 cells (*n* = 8). **e**, Th2 and Th17 cells were increased by olaparib but unaffected by CCR1/5 antagonists BX plus MAV in FACS analyses of bone marrow cells from B6 mice intracardiacally injected with Py8119 cells (*n* = 10). **f**, **g**, Neither CCL3 overexpression in Py8119 (**f**, *n* = 4) nor CCR1/5 antagonists BX plus MAV (**g**, *n* = 6) affected olaparib-induced IMC accumulation in bone marrow. Data represent mean ± SEM, **P* < 0.05, ***P* < 0.01, ****P* < 0.001, *****P* < 0.0001; n.s., non-significant. Two-sided Student's *t*-test was used to calculate statistical difference. Source data are provided as a Source Data file.

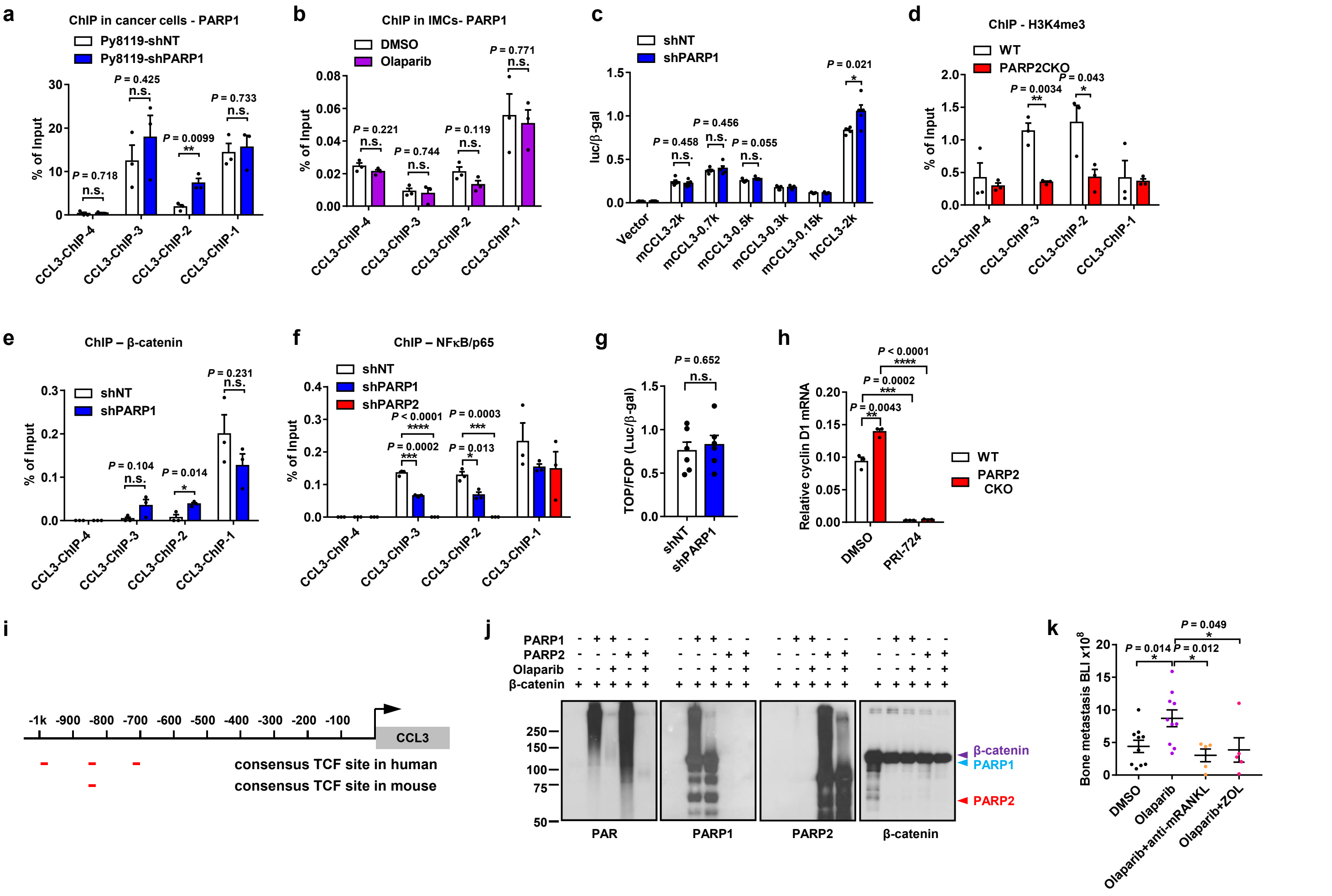

**Supplementary Fig. 7 | PARP1 does not bind to CCL3 promoter or β-catenin transcription factor. a**, ChIP analyses of PARP1KD Py8119 cells vs. control cells showed that PARP1 did not associate with CCL3 promoter (*n* = 3). **b**, Olaparib (3 μM) did not affect PARP1 signal at CCL3 promoter in IMCs (*n* = 3). **c**, PARP1KD did not affect CCL3 promoter driven luciferase expression (*n* = 5). **d**, ChIP analyses showed that H3K4me3 (Histone H3 trimethyl Lys4) level at CCL3 promoter was lower in PARP2CKO IMCs compared with WT control IMCs (*n* = 3). **e**, In ChIP, PARP1KD did not alter β-catenin binding to CCL3 promoter in Py8119 cells (*n* = 3). **f**, ChIP of p65/NFκB showed that both PARP1KD and PARP2KD reduced p65/NFκB binding to CCL3 promoter (*n* = 3). **g**, TOP/FOP flash assay revealed that PARP1 did not regulate β-catenin transcriptional activity (*n* = 6). **h**, Treatment with β-catenin inhibitor PRI-724 eliminated the expression of β-catenin target gene cyclin D1 (*n* = 3). **i**, Consensus TCF binding sites CTTTG(A/T)(A/T) identified at human or mouse CCL3 promoter. **j**, *In vitro* PARylation assay with recombinant human PARP1, PARP2 and GST-β-catenin with or without olaparib showed no PARylation of β-catenin. Western blot analyses were repeated twice and showed similar results. **k**, Both anti-RANKL and zoledronate (ZOL) (*n* = 5) diminished olaparib-induced bone metastasis (*n* = 10). Data represent mean ± SEM, *$P < 0.05$, **$P < 0.01$, ***$P < 0.001$, ****$P < 0.0001$; n.s., non-significant. Two-sided Student's *t*-test was used to calculate statistical difference. Source data are provided as a Source Data file.

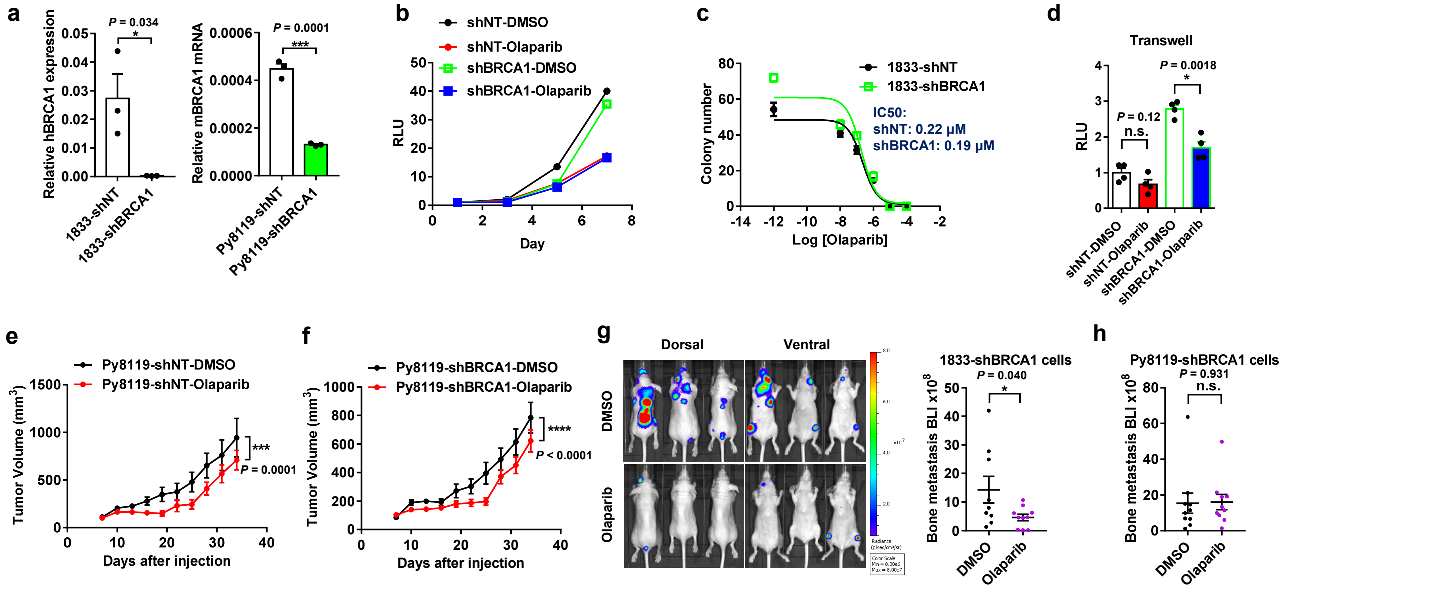


**Supplementary Fig. 8 | Olaparib suppresses primary tumor growth and bone metastasis of BRCA1-deficient breast cancer cells.** **a**, RT-qPCR showing the efficiency of stable knockdown of BRCA1 in 1833 (~100%) and Py8119 (~70%) cells ($n$ = 3). **b**, 10 μM Olaparib had similar suppressive effect on the proliferation of 1833-shNT and 1833-shBRCA1 cells ($n$ = 6). **c**, Clonogenic assay showed similar IC50s of olaparib to the survival of 1833-shNT and 1833-shBRCA1 cells ($n$ = 4). **d**, 10 μM Olaparib significantly suppressed the migration of 1833-shBRCA1 cells but not control cells ($n$ = 4). **e**, **f**, Olaparib similarly suppressed primary tumor growth of Py8119-shNT cells (**e**, $n$ = 10) or Py8119-shBRCA1 cells (**f**, $n$ = 10) at mammary fat pad. Statistical analysis was performed using two-way ANOVA with Sidak's multiple comparison. **g**, Bone metastasis of 1833-shBRCA1 cells was reduced by olaparib treatment ($n$ = 10). Left, BLI images; right, BLI signals. **h**, Bone metastasis of Py8119-shBRCA1 cells was not altered by olaparib treatment ($n$ = 10). Data represent mean ± SEM, *$P$ < 0.05, **$P$ < 0.01, ***$P$ < 0.001, ****$P$ < 0.0001; n.s., non-significant. **a**, **d**, **g**, **h**, Two-sided Student's $t$-test was used to calculate statistical difference. Source data are provided as a Source Data file.

**a**

| Identifier | Other Name | Cancer types | Inhibitor name | PARP inhibitor | | | | Physician's Choice Treatment or Placebo | | | | Refer-ence |
|---|---|---|---|---|---|---|---|---|---|---|---|---|
| | | | | Fracture | Serious bone or musculoskeletal pain | Non-serious bone or musculoskeletal pain | Osteolysis or Osteonecrosis | Fracture | Serious bone or musculoskeletal pain | Non-serious bone or musculoskeletal pain | Osteolysis or Osteonecrosis | |
| NCT00494234 | ICEBERG 1 | Breast | Olaparib | n/a | 1/54 (1.85%) | 7/54 (12.96%) | n/a | n/a | n/a | n/a | n/a | 1 |
| NCT00494442 | ICEBERG 2 / study 9 | Ovarian | Olaparib | 1/57 (1.75%) | n/a | n/a | n/a | n/a | n/a | n/a | n/a | 2 |
| NCT00628251 | ICEBERG 3 / study 12 | Ovarian | Olaparib | n/a | 1/64 (1.56%) | 4/64 (6.25%) | n/a | n/a | 0/32 (0.00%) | 4/32 (12.5%) | n/a | 3 |
| NCT00753545 | study 19 | Ovarian | Olaparib | 3/126 (2.38%) | n/a | 10/136 (7.35%) | 1/136 (0.74%) | 0/128 (0.00%) | n/a | 8/128 (6.25%) | 0/128 (0.00%) | 4 |
| NCT00679783 | study 20 | Ovarian/Breast | Olaparib | n/a | n/a | 5/90 (5.56%) | n/a | n/a | n/a | n/a | n/a | 5 |
| NCT01078662 | study 42 | Ovarian/ Breast/ Prostate/ Pancreatic/ Advanced Tumours | Olaparib | 1/298 (0.34%) | n/a | 32/298 (10.74%) | 1/298 (0.34%) | n/a | n/a | n/a | n/a | 6 |
| NCT02000622 | OlympiAD | Breast | Olaparib | 2/205 (0.98%) | n/a | 24/205 (11.71%) | n/a | 0/91 (0.00%) | n/a | 10/91 (10.99%) | n/a | 7 |
| NCT01844986 | SOLO-1 | Ovarian | Olaparib | 0/260 (0.00%) | n/a | 16/260 (6.15%) | n/a | 1/130 (0.77%) | n/a | 11/130 (8.46%) | n/a | 8 |
| NCT01874353 | SOLO-2 | Ovarian | Olaparib | n/a | n/a | 7/195 (3.59%) | n/a | n/a | n/a | 6/99 (6.06%) | n/a | 9 |
| NCT01945775 | EMBRACA | Breast | Talazoparib | 7/286 (2.45%) | 3/286 (1.05%) | 68/286 (23.78%) | 3/286 (0.70%) | 1/126 (0.79%) | 0/126 (0.00%) | 24/126 (19.05%) | 0/126 (0.00%) | 10 |
| NCT02034916 | ABRAZO | Breast | Talazoparib | n/a | n/a | 9/83 (10.84%) | n/a | n/a | n/a | n/a | n/a | 11 |
| NCT01891344 | ARIEL2 | Ovarian | Rucaparib | n/a | n/a | n/a | n/a | n/a | n/a | n/a | n/a | 12 |
| NCT01968213 | ARIEL3 | Ovarian | Rucaparib | 4/372 (1.08%) | n/a | n/a | n/a | 2/189 (1.06%) | n/a | n/a | n/a | 13 |
| NCT01847274 | ENGOT-OV16/NOVA | Ovarian | Niraparib | n/a | n/a | 20/367 (5.45%) | n/a | n/a | n/a | 5/179 (2.79%) | n/a | 14 |

**b**

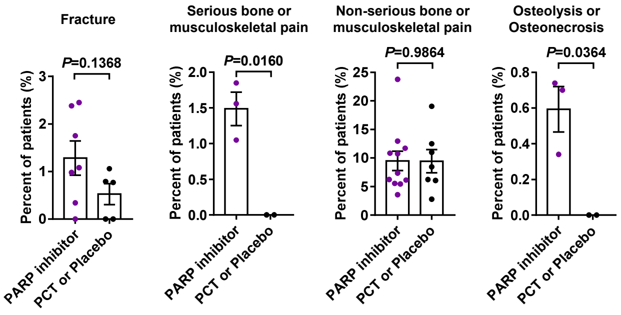


**Supplementary Fig. 9 | Increased incidence of bone health related adverse events in PARP inhibitor treated patients. a**, A summary of available data on adverse events related to bone health in PARP inhibitor monotherapy clinical trials. The clinical results were from https://clinicaltrials.gov/ and published articles. Reported data is presented as adverse events / total patient number (event incidence) in the group for PARP inhibitor treatment and the group for Physician's Choice Treatment or Placebo when available. **b**, Statistic analyses illustrate that patients with PARP inhibitor treatment showed increased incidence of serious bone or musculoskeletal pain and osteolysis/osteonecrosis, as well as an increased risk of bone fracture. The incidence of non-serious bone or musculoskeletal pain are comparable between the two groups of patients. Data represent mean ± SEM. Two-sided Student's *t*-test was used to calculate statistical difference. n/a, not available.

**Supplementary Fig. 10 The development of the BLI signal over the entire course in main and Supplementary figures (Suppl.).**

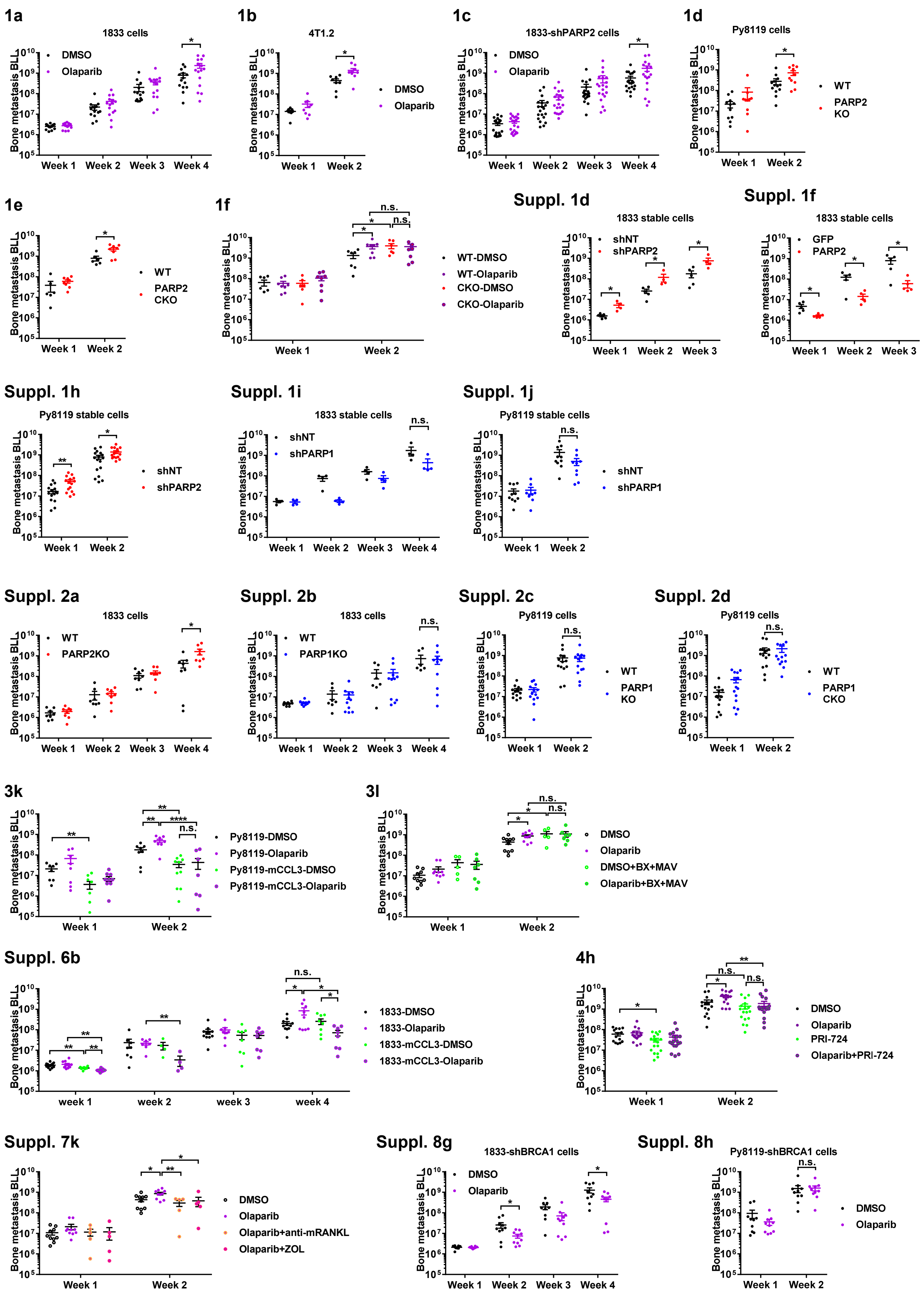

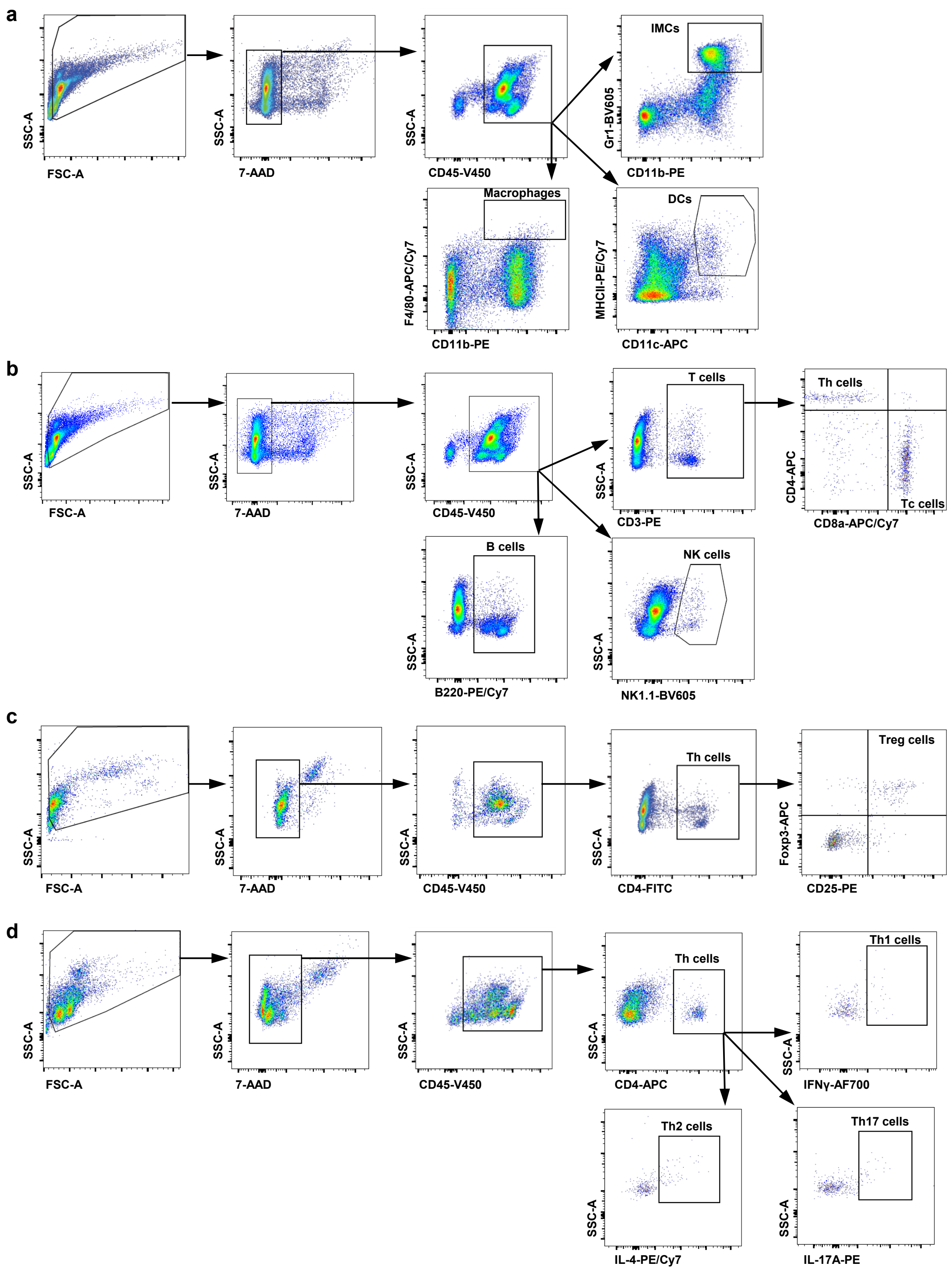


**Supplementary Fig. 11 | Gating strategies used for FACS analyses. a**, Gating strategies for CD11b+ Gr1+ IMCs, CD11b+ F4/80+ macrophages, and CD11c+ MHCII+ DCs in CD45+ mouse bone marrow cells presented on Fig. 3a-c and Supplementary Fig. 5a, 5c-d, 6c, 6f-g. **b**, Gating strategies for B220+ B cells, NK1.1 NK cells, and CD3+ T cells in CD45+ mouse bone marrow cells, and for CD4+ Th cells and CD8a+ Tc cells in CD3+ T cells presented on Supplementary Fig. 5b-c, 6c. **c**, Gating strategies for CD25+ Foxp3+ Treg cells in CD4+ Th cells from mouse bone marrow presented on Fig. 3m-n. **d**, Gating strategies for IFNγ+ Th1 cells, IL-4+ Th2 cells, and IL-17A+ Th17 cells in CD4+ Th cells from mouse bone marrow presented on Fig. 3m-n and Supplementary Fig. 6d-e.

**Supplementary Table 1. RAW data of PARP2 regulated genes from RNA-seq of PARP1WT vs. PARP1CKO and PARP2WT vs. PARP2CKO.**

| GENE_NAME | FPKM (Fragments Per Kilobase of exon model per Million mapped fragments) | | | | | | | | PARP1WT v.s. PARP1CKO | | | PARP2WT v.s. PARP2CKO | | |
|---|---|---|---|---|---|---|---|---|---|---|---|---|---|---|
| | PARP1WT | | PARP1CKO | | PARP2WT | | PARP2CKO | | $log_2FC$ | P value | FDR | $log_2FC$ | P value | FDR |
| Ifi202b | 0 | 0 | 0 | 0.28667534 | 19.9229723 | 19.7773186 | 0 | 0 | 5.80006401 | 4.45E-05 | 0.01165844 | -12.961331 | 1.88E-188 | 3.30E-184 |
| Nlrp1c-ps | 8.15231213 | 8.52532977 | 9.99637519 | 8.68846801 | 3.51683772 | 6.15636378 | 0 | 0 | 0.16396938 | 0.43747902 | 0.89988942 | -10.921348 | 1.59E-69 | 1.39E-65 |
| Fmr1nb | 0.12899228 | 0.24185333 | 0.07933631 | 0 | 0.2945135 | 0.26934092 | 0.01680561 | 0 | -2.1310336 | 0.00422302 | 0.24831141 | -4.6006639 | 8.95E-08 | 9.81E-05 |
| Xlr3b | 0.56756603 | 0.90694998 | 0.63469049 | 0.63950653 | 0.19056756 | 0.25010228 | 0.01680561 | 0 | -0.2307358 | 0.51403366 | 0.94076369 | -4.238461 | 1.88E-06 | 0.00150011 |
| Rmrp | 0.05159691 | 0.04030889 | 0.03966816 | 0.0882078 | 8.64483755 | 0.7310682 | 0.47055717 | 0.12404127 | 0.45346524 | 0.68763444 | 0.98535912 | -3.9520249 | 2.96E-06 | 0.00216618 |
| Spint1 | 5.03069894 | 5.38123652 | 4.00648371 | 5.88787046 | 2.97978369 | 0.17314773 | 0.18486175 | 0.16538836 | -0.0739577 | 0.90931038 | 1 | -3.153011 | 2.88E-05 | 0.01681118 |
| Wfdc18 | 0.07739537 | 0.16123555 | 0.09917039 | 0.11025975 | 0.25986486 | 0.42325001 | 0.01680561 | 0.06202064 | -0.2361237 | 0.728908 | 0.99709522 | -3.1031098 | 2.58E-06 | 0.00196924 |
| Nlrp1a | 22.4446568 | 19.3079573 | 22.4323419 | 19.979066 | 9.56302671 | 17.8726936 | 2.08389606 | 1.73657783 | 0.02354476 | 0.90754751 | 1 | -2.8364624 | 1.05E-34 | 4.61E-31 |
| Hdc | 7.97172294 | 8.76718309 | 8.60798974 | 11.0700785 | 5.5437836 | 0.98117048 | 0.80666944 | 0.86828891 | 0.23287449 | 0.60552596 | 0.96495836 | -1.962622 | 5.55E-05 | 0.02952358 |
| Ccl6 | 104.070972 | 95.9351529 | 93.3193358 | 127.239747 | 54.1731604 | 104.081025 | 22.5195219 | 20.4461366 | 0.14108917 | 0.49285568 | 0.93389947 | -1.880297 | 1.45E-18 | 4.25E-15 |
| Wfdc17 | 11.764096 | 20.476915 | 11.5632673 | 13.6722086 | 38.3907014 | 67.7777174 | 16.4022787 | 14.8436057 | -0.3563204 | 0.1319374 | 0.76123149 | -1.7638378 | 1.70E-13 | 3.31E-10 |
| Egr1 | 72.3388709 | 51.6558397 | 14.4590427 | 7.14483157 | 41.0586473 | 79.1669904 | 17.5954772 | 22.5341647 | -2.5191445 | 1.41E-16 | 2.74E-13 | -1.5831279 | 7.35E-08 | 8.59E-05 |
| Kcnj10 | 0.4127753 | 0.46355221 | 0.39668156 | 0.50719483 | 1.62848643 | 1.38518185 | 0.67222454 | 0.37212382 | 0.03144819 | 0.93573702 | 1 | -1.5016775 | 1.42E-06 | 0.00118682 |
| Fosb | 6.81079241 | 6.44942205 | 0.93220165 | 0.61745458 | 1.73243238 | 4.69422738 | 1.39486591 | 0.9509831 | -3.0874727 | 1.72E-15 | 3.02E-12 | -1.4420894 | 8.06E-05 | 0.0392966 |
| Slfn8 | 27.888131 | 22.1295794 | 25.1892787 | 35.3272227 | 13.3743779 | 19.9312277 | 7.74738777 | 6.86361712 | 0.27550015 | 0.12241772 | 0.74928527 | -1.1860281 | 1.70E-10 | 2.98E-07 |
| Ccl4 | 71.2037388 | 70.4800903 | 64.1037393 | 72.3303936 | 47.1741336 | 80.1289223 | 26.5696747 | 34.8555978 | -0.0545451 | 0.75469319 | 1 | -1.0517389 | 2.88E-09 | 4.22E-06 |
| Ccl9 | 528.687761 | 531.371913 | 506.820189 | 480.401715 | 393.054257 | 554.553706 | 234.774419 | 235.74044 | -0.1026881 | 0.37308049 | 0.86146873 | -1.0099683 | 4.49E-18 | 9.86E-15 |
| Ccl3 | 135.493491 | 131.991453 | 138.124517 | 143.469982 | 104.985402 | 146.329071 | 62.7017435 | 63.8605822 | 0.07419271 | 0.49083519 | 0.93224673 | -0.9893628 | 1.29E-19 | 4.54E-16 |
| Parp2 | 38.9814672 | 40.77244 | 38.2797701 | 36.8708592 | 48.265566 | 48.3659329 | 25.8134221 | 25.5938494 | -0.0864414 | 0.19969231 | 0.77375933 | -0.9102434 | 1.17E-41 | 6.83E-38 |
| Scd1 | 149.656844 | 85.2331432 | 107.004849 | 144.748995 | 136.238482 | 140.865299 | 66.7014795 | 89.847229 | 0.10042777 | 0.61302389 | 0.96495836 | -0.8240635 | 3.74E-05 | 0.02113605 |
| Hmga2-ps1 | 9.54542876 | 8.50517532 | 9.32201654 | 7.6079225 | 16.7699454 | 15.8333981 | 9.99933996 | 9.34444259 | -0.086695 | 0.49729858 | 0.93552779 | -0.7520351 | 3.28E-10 | 5.23E-07 |
| Lyz2 | 5701.58779 | 6387.00373 | 3496.21239 | 4320.77073 | 9742.61065 | 10813.2297 | 6758.59589 | 5736.78485 | -0.6289721 | 1.40E-06 | 0.00066275 | -0.7181479 | 3.72E-08 | 4.66E-05 |
| Retnlg | 19.9422066 | 19.4087295 | 17.4738225 | 18.1046504 | 9.85754022 | 12.9860798 | 7.61294286 | 7.25641449 | -0.1451894 | 0.21457271 | 0.77375933 | -0.6157784 | 7.79E-07 | 0.00075912 |
| Slfn10-ps | 23.4507966 | 23.7419349 | 24.0587363 | 23.9925208 | 18.0519454 | 21.7973755 | 13.5117132 | 13.1690485 | 0.02579324 | 0.78958736 | 1 | -0.5766734 | 6.28E-09 | 8.47E-06 |
| Cxcr2 | 19.7616174 | 19.8521272 | 19.2787236 | 24.5217676 | 13.4609996 | 16.3913186 | 10.7387869 | 10.4401405 | 0.14361848 | 0.23848778 | 0.77375933 | -0.4933961 | 8.56E-05 | 0.04059047 |
| Lyz1 | 258.629522 | 252.857653 | 149.886126 | 183.251698 | 411.002257 | 462.631499 | 336.935743 | 303.96314 | -0.6187595 | 2.99E-11 | 3.40E-08 | -0.4467733 | 1.29E-06 | 0.00116878 |
| Mmp9 | 46.6436086 | 48.5520553 | 46.9670961 | 56.0340031 | 29.2607828 | 32.5710121 | 24.4689731 | 21.4384667 | 0.11261606 | 0.22658698 | 0.77375933 | -0.4272614 | 8.62E-06 | 0.00560069 |
| Phgdh | 115.2933 | 88.0346109 | 98.5753664 | 108.892526 | 82.3078622 | 91.5374339 | 60.9539597 | 68.6361712 | 0.02992738 | 0.77805241 | 1 | -0.4241433 | 7.02E-05 | 0.03516416 |
| Fads2 | 145.322703 | 123.788594 | 124.756349 | 149.115281 | 137.918941 | 134.747412 | 98.5145056 | 107.626478 | 0.02565224 | 0.77489782 | 1 | -0.4038356 | 6.85E-06 | 0.00461992 |
| Chd3 | 46.6178102 | 43.6948344 | 62.8541924 | 69.1769649 | 52.3021334 | 58.6778422 | 46.2826592 | 37.6672 | 0.54825632 | 3.66E-09 | 2.47E-06 | -0.4006909 | 1.49E-05 | 0.00902234 |
| Pck2 | 39.2652502 | 34.9881146 | 39.0731332 | 38.1498722 | 34.4927286 | 36.4572167 | 27.7628733 | 29.8112527 | 0.0588702 | 0.45132647 | 0.90751476 | -0.3021303 | 0.00011208 | 0.04915098 |
| Tfrc | 378.334359 | 385.534357 | 461.776998 | 481.614572 | 531.423631 | 498.203739 | 673.955513 | 593.640861 | 0.30447088 | 8.87E-05 | 0.01945385 | 0.30003887 | 0.00010627 | 0.04779889 |
| Atp6v0d2 | 67.0759859 | 64.7562282 | 57.0626417 | 55.7252758 | 106.475294 | 127.186628 | 146.360087 | 142.151299 | -0.2246033 | 0.00438717 | 0.25370098 | 0.304679 | 6.05E-05 | 0.03120391 |
| Plscr3 | 37.2013737 | 39.0391578 | 41.1358773 | 39.5391451 | 46.2559444 | 41.20916 | 54.7190772 | 57.5758244 | 0.0808945 | 0.27221404 | 0.78526208 | 0.35922645 | 4.34E-07 | 0.00044831 |
| Pdpn | 6.47541248 | 7.45714424 | 6.5055775 | 6.83610428 | 16.215567 | 15.4486253 | 25.4941155 | 19.2677445 | -0.0671047 | 0.64354841 | 0.9709298 | 0.50132168 | 9.48E-05 | 0.04375196 |
| Dusp23 | 10.4999716 | 11.7298863 | 12.0591193 | 11.5111175 | 7.70932407 | 8.54195474 | 11.8815687 | 11.1223675 | 0.08185166 | 0.47636276 | 0.92319852 | 0.50381655 | 1.22E-05 | 0.00765151 |
| Folr2 | 30.4937751 | 26.7852559 | 45.5985448 | 43.7069635 | 24.4619451 | 29.8583643 | 42.770286 | 35.1450274 | 0.64241943 | 3.49E-09 | 2.45E-06 | 0.52253691 | 1.33E-06 | 0.00116878 |
| Slamf7 | 128.089335 | 118.367049 | 137.926177 | 143.734605 | 94.2269969 | 88.8440247 | 139.385757 | 132.165977 | 0.19313631 | 0.00316025 | 0.21759945 | 0.56886802 | 4.16E-18 | 9.86E-15 |
| Sel1l3 | 5.44347424 | 5.09907431 | 6.6840842 | 7.03457182 | 5.26659442 | 4.94432966 | 9.78086699 | 6.71890231 | 0.38097136 | 0.02985455 | 0.54401419 | 0.69616032 | 4.58E-05 | 0.02512967 |
| 2410003L11Rik | 0.33537993 | 0.08061778 | 0.25784301 | 0.19846754 | 0.08662162 | 0.03847727 | 0.55458524 | 0.35145027 | 0.20685646 | 0.72553005 | 0.99670761 | 2.79827498 | 6.13E-06 | 0.00430134 |

Red color labels immune chemokines.

## Supplementary Table 2. Primer sequences

| Primers | Sequences | Primers | Sequences |
|---|---|---|---|
| mL19 qPCR | GGTCTGGTTGGATCCCAATG<br>CCCATCCTTGATCAGCTTCCT | mPARP2 qPCR | ATCTTCGAGTCCAGGAGCTG<br>ACTGTCAGCTTTCCAAGCG |
| mTRAP qPCR | AAGTATGCCCACACCAACTGATC<br>GAAAGCCCGTTCCCAAGAAA | hPARP1 qPCR | GGCTTAATCCTGTTGGGAGA<br>CCAAACCTTTGACACTGTGC |
| mCTSK qPCR | AGCAGGCTGGAGGACTAAGGT<br>GATTTGTGCATCTCAGTGGAAGAC | hPARP2 qPCR | GCATGACTTTGGACTCCGTA<br>GCAATTTCAATGTCTCCCAA |
| mCCL3 qPCR | GTGGAATCTTCCGGCTGTAG<br>ACCATGACACTCTGCAACCA | mRANKL qPCR | TCCATGTTCCTGGCCCTCCT<br>AGGATCCATCTGCGCTCGAA |
| mCCL4 qPCR | GAAACAGCAGGAAGTGGGAG<br>CATGAAGCTCTGCGTGTCTG | mOPG qPCR | AGGGCGTTACCTGGAGATCG<br>TGCAAACTGTGTTTCGCTCTGG |
| mCCL6 qPCR | TTGGAGGGTTATAGCGACGA<br>CCAGGAGGATGAGAAACTCC | PyMT qPCR | CCAACAGATACACCCGCACAT<br>GGTCTTGGTCGCTTTCTGGATA |
| mCCL9 qPCR | GCCTTCAGACTGCTCTGGAC<br>ACTGCCCTCTCCTTCCTCAT | mVimentin qPCR | AGCTGCTAACTACCAGGACACTATTG<br>CGAAGGTGACGAGCCATCTC |
| mCCL3-ChIP-1 | GCCCCTTCTCTTATAGGTAGCCC<br>CACTGTGGACCCTGGGTTG | Luciferase qPCR | CAACTGCATAAGGCTATGAAGAGA<br>ATTTGTATTCAGCCCATATCGTTT |
| mCCL3-ChIP-2 | GTCTCAGCTCTCAACTCGTGACC<br>TTCTCATGAGATGGGACCAGC | mCAR2 qPCR | TGGTCAACTTAGGGCATCTTTTC<br>TCCTATGGCTGTGAAGAGAAGCA |
| mCCL3-ChIP-3 | CTCAGCTTCTCTAACCACATGGA<br>GACCGCGTCTTCCCCATT | mOscar qPCR | AATGCTTTGCCTGTATGCATGTA<br>TTCCAAGAGATCAGATGCCTTCT |
| mCCL3-ChIP-4 | GTGACTAGGCCACTGGGTTGA<br>CTTTCCTAGAAAGGCTATTTCTAGCTT | mCalcr qPCR | TGCCTTTCAGAGGTGATTTAGTTG<br>CTCAGTATCCAAGGTCCACAGTTG |
| mCCL3-promoter-2k<br>mCCL3-promoter-0.7k | AGAAAGGGACACATTATAGGAA<br>GAGATGGCTTTACATTTGGG | mNFATc1 qPCR | AGTCTCTTTCCCCGACATCA<br>GATCCGAAGCTCGTATGGAC |
| mCCL3-promoter-0.5k<br>mCCL3-promoter-0.3k | GGCTGATGATTGGACAAATG<br>TTATTCACAGAACGGTTCTCA | mRANK qPCR | TCGGAGCTCAGCATCCCTTG<br>TCCAAGGAGGGTGCAGTTGG |
| mCCL3-promoter-0.15k<br>mCCL3-promoter+75 | GATGCTATTCTTAGATATCCTGG<br>GCTTGTCAGCAGAACAGAA | mCyclin D1 qPCR | TCCCTAGCAAGCTGCCAAAC<br>TGGACCCACCACCAGTCTATG |
| hCCL3-promoter-2k<br>hCCL3-promoter+75 | CTACAGACACTTAGAAAGGACAGA<br>CTCGAGTGTCAGCAGAGC | hBRCA qPCR | TGTGAAGGCCCTTTCTTCTG<br>TCCCATCTGTCTGGAGTTGA |
| hGAPDH qPCR | AAGGTGAAGGTCGGAGTCAA<br>AATGAAGGGGTCATTGATGG | mBRCA qPCR | TGTGGTCACACTTTGTGGAAA<br>ATCTGCCGTCCAAATTCAAG |
| mPARP1 qPCR | CTGTCCAAGAAGATGGTGGA<br>CTTGAGCTGACTGGCACTGT | | |

**Supplementary References**


1. Tutt, A., *et al.* Oral poly(ADP-ribose) polymerase inhibitor olaparib in patients with BRCA1 or BRCA2 mutations and advanced breast cancer: a proof-of-concept trial. *Lancet* **376**, 235-244 (2010).
2. Audeh, M.W., *et al.* Oral poly(ADP-ribose) polymerase inhibitor olaparib in patients with BRCA1 or BRCA2 mutations and recurrent ovarian cancer: a proof-of-concept trial. *Lancet* **376**, 245-251 (2010).
3. Kaye, S.B., *et al.* Phase II, open-label, randomized, multicenter study comparing the efficacy and safety of olaparib, a poly (ADP-ribose) polymerase inhibitor, and pegylated liposomal doxorubicin in patients with BRCA1 or BRCA2 mutations and recurrent ovarian cancer. *Journal of clinical oncology : official journal of the American Society of Clinical Oncology* **30**, 372-379 (2012).
4. Ledermann, J.A., *et al.* Overall survival in patients with platinum-sensitive recurrent serous ovarian cancer receiving olaparib maintenance monotherapy: an updated analysis from a randomised, placebo-controlled, double-blind, phase 2 trial. *The Lancet. Oncology* **17**, 1579-1589 (2016).
5. Gelmon, K.A., *et al.* Olaparib in patients with recurrent high-grade serous or poorly differentiated ovarian carcinoma or triple-negative breast cancer: a phase 2, multicentre, open-label, non-randomised study. *The Lancet. Oncology* **12**, 852-861 (2011).
6. Kaufman, B., *et al.* Olaparib monotherapy in patients with advanced cancer and a germline BRCA1/2 mutation. *Journal of clinical oncology : official journal of the American Society of Clinical Oncology* **33**, 244-250 (2015).
7. Robson, M., *et al.* Olaparib for Metastatic Breast Cancer in Patients with a Germline BRCA Mutation. *N Engl J Med* **377**, 523-533 (2017).
8. Moore, K., *et al.* Maintenance Olaparib in Patients with Newly Diagnosed Advanced Ovarian Cancer. *The New England journal of medicine* **379**, 2495-2505 (2018).
9. Friedlander, M., *et al.* Health-related quality of life and patient-centred outcomes with olaparib maintenance after chemotherapy in patients with platinum-sensitive, relapsed ovarian cancer and a BRCA1/2 mutation (SOLO2/ENGOT Ov-21): a placebo-controlled, phase 3 randomised trial. *The Lancet. Oncology* **19**, 1126-1134 (2018).
10. Litton, J.K., *et al.* Talazoparib in Patients with Advanced Breast Cancer and a Germline BRCA Mutation. *The New England journal of medicine* **379**, 753-763 (2018).
11. Turner, N.C., *et al.* A Phase II Study of Talazoparib after Platinum or Cytotoxic Nonplatinum Regimens in Patients with Advanced Breast Cancer and Germline BRCA1/2 Mutations (ABRAZO). *Clinical cancer research : an official journal of the American Association for Cancer Research* **25**, 2717-2724 (2019).
12. Swisher, E.M., *et al.* Rucaparib in relapsed, platinum-sensitive high-grade ovarian carcinoma (ARIEL2 Part 1): an international, multicentre, open-label, phase 2 trial. *The Lancet. Oncology* **18**, 75-87 (2017).
13. Coleman, R.L., *et al.* Rucaparib maintenance treatment for recurrent ovarian carcinoma after response to platinum therapy (ARIEL3): a randomised, double-blind, placebo-controlled, phase 3 trial. *Lancet* **390**, 1949-1961 (2017).
14. Mirza, M.R., *et al.* Niraparib Maintenance Therapy in Platinum-Sensitive, Recurrent Ovarian Cancer. *The New England journal of medicine* **375**, 2154-2164 (2016).
