# Supplementary material for: Differential regulation of breast cancer bone metastasis by PARP1 and PARP2
Source: Nat Commun. 2020 Mar 27;11:1578. doi: 10.1038/s41467-020-15429-z (PMC7101362; doi:10.1038/s41467-020-15429-z)
Supplement: Supplementary file 3 — Reporting Summary [file 41467_2020_15429_MOESM3_ESM.pdf]

nature research

Corresponding author(s): Yihong Wan

Last updated by author(s): Feb 21, 2020

Reporting Summary

Nature Research wishes to improve the reproducibility of the work that we publish. This form provides structure for consistency and transparency in reporting. For further information on Nature Research policies, see Authors & Referees and the Editorial Policy Checklist .

Statistics

For all statistical analyses, confirm that the following items are present in the figure legend, table legend, main text, or Methods section.

n/a	Confirmed	
☐	☒	The exact sample size (*n*) for each experimental group/condition, given as a discrete number and unit of measurement
☐	☒	A statement on whether measurements were taken from distinct samples or whether the same sample was measured repeatedly
☐	☒	The statistical test(s) used AND whether they are one- or two-sided<br>*Only common tests should be described solely by name; describe more complex techniques in the Methods section.*
☒	☐	A description of all covariates tested
☐	☒	A description of any assumptions or corrections, such as tests of normality and adjustment for multiple comparisons
☐	☒	A full description of the statistical parameters including central tendency (e.g. means) or other basic estimates (e.g. regression coefficient) AND variation (e.g. standard deviation) or associated estimates of uncertainty (e.g. confidence intervals)
☐	☒	For null hypothesis testing, the test statistic (e.g. *F*, *t*, *r*) with confidence intervals, effect sizes, degrees of freedom and *P* value noted<br>*Give P values as exact values whenever suitable.*
☒	☐	For Bayesian analysis, information on the choice of priors and Markov chain Monte Carlo settings
☒	☐	For hierarchical and complex designs, identification of the appropriate level for tests and full reporting of outcomes
☒	☐	Estimates of effect sizes (e.g. Cohen's *d*, Pearson's *r*), indicating how they were calculated

Our web collection on statistics for biologists contains articles on many of the points above.

Software and code

Policy information about availability of computer code

Data collection	BD FACSDiva software Version 8.0.1
Data analysis	Graphpad Prism verson 7, Scanco, Bioquant version 14.1.6, FastQC version 0.11.2, FastQ Screen version 0.4.4, fastq-mcf ea-utils/verson 1.1.2-806, TopHat verson 2.0.12, Picard tools verson 1.127, Subread/FeatureCounts, edgeR, ImageJ version 1.4.3.67.

For manuscripts utilizing custom algorithms or software that are central to the research but not yet described in published literature, software must be made available to editors/reviewers. We strongly encourage code deposition in a community repository (e.g. GitHub). See the Nature Research guidelines for submitting code & software for further information.

Data

Policy information about availability of data

All manuscripts must include a data availability statement. This statement should provide the following information, where applicable:

- Accession codes, unique identifiers, or web links for publicly available datasets
- A list of figures that have associated raw data
- A description of any restrictions on data availability

The RNA-seq data have been deposited in the GEO database under the accession code XX. Clinical data are available in Clinicaltrials.gov with Identifier NCT00494234, NCT00494442, NCT00628251, NCT00753545, NCT00679783, NCT01078662, NCT02000622, NCT01844986, NCT01874353, NCT01945775, NCT02034916, NCT01891344, NCT01968213, and NCT01847274. The source data underlying Figs 1a-f, 2b-d, 2f-h, 3a-c, 3h-n, 4a-h and Supplementary Figs 1a, 1c-1n, 1p-q, 2a-p, 3a-b, 3d-f, 3h-k, 4a-f, 4h, 5c-g, 6a-g, 7a-h, 7j-k, and 8a-h are provided as a Source Data file. All the other data supporting the findings of this study are available within the article and its supplementary information files and from the corresponding author upon reasonable request. A reporting summary for this article is available as a Supplementary Information file.

Field-specific reporting

Please select the one below that is the best fit for your research. If you are not sure, read the appropriate sections before making your selection.

- [x] Life sciences
- [ ] Behavioural & social sciences
- [ ] Ecological, evolutionary & environmental sciences

For a reference copy of the document with all sections, see nature.com/documents/nr-reporting-summary-flat.pdf

Life sciences study design

All studies must disclose on these points even when the disclosure is negative.

Sample size	Sample size estimate was based on power analyses performed using SAS 9.3 TS X64_7PRO platform. For bone and bone metastasis analyses, at type I error rate of 0.05 (two-sided test), a sample size of n=5 will provide >90% power. Thus, each experimental group will consist of at least 5 mice to yield statistically meaningful results.
Data exclusions	Mice with massive BLI signals (>10^10) on chest only were excluded as failed intracardiac injections.
Replication	Unless stated, all data shown were obtained from at least 3 biological independent experiments. Western blot analyses were independently repeated at least twice and showed similar results. H&E staining of bone metastases were repeated independently twice and showed similar results. Two biological independent pairs of mouse litermates were used for RNA-seq.
Randomization	Animal/Sample allocation was random.
Blinding	RNA-seq was carried out as a blinded experiment. No blinding was performed for other experiments, as animal genotypes were first analyzed. Random animal subgroup allocation and the same standard experimental procedures on animals are performed to eliminate random errors. Standard protocols were equally applied on all samples.

Reporting for specific materials, systems and methods

We require information from authors about some types of materials, experimental systems and methods used in many studies. Here, indicate whether each material, system or method listed is relevant to your study. If you are not sure if a list item applies to your research, read the appropriate section before selecting a response.

Materials & experimental systems

n/a	Involved in the study	
[ ]	[x]	Antibodies
[ ]	[x]	Eukaryotic cell lines
[x]	[ ]	Palaeontology
[ ]	[x]	Animals and other organisms
[x]	[ ]	Human research participants
[x]	[ ]	Clinical data

Methods

n/a	Involved in the study	
[x]	[ ]	ChIP-seq
[ ]	[x]	Flow cytometry
[x]	[ ]	MRI-based neuroimaging

Antibodies

Antibodies used	Polyclonal antibodies for PARP1 (Catalog#39559) and PARP2 (#39743) were purchased from Active Motif (Carlsbad, CA). Antibody for β-actin (clone AC-15, #A5441) was from Sigma-Aldrich (St. Louis, MO). Antibodies for β-catenin (clone E5, #sc-7963) and p65 (clone F-6, #sc-8008) were from Santa Cruz Biotechnology (Dallas, TX). Polyclonal antibody for H3K4me3 (ab8580) was from Abcam (Cambridge, MA). Anti-poly-ADP-ribose binding reagent (Anti-PAR) (#MABE1031) was from MilliporeSigma (Burlington, MA). Anti-CD45 (clone 30-F11, #BDB560501) was from BD Biosciences (Franklin Lakes, NJ). Anti-FoxP3 (clone FJK-16s, #17-5773-82) was from Invitrogen (Carlsbad, CA). The following antibodies were from Biolegend (San Diego, CA): antibodies against CD16/32 (clone 93, Cat#101301), CD11b (M1/70, #101207), F4/80 (BM8, #123117), CD11c (N418, #117309), MHC II (M5/114.15.2, #107629), Gr1 (RB6-8C5, #108440), B220 (RA3-6B2, #103221), NK1.1 (PK136), CD3 (17A2, #100205), CD4 (RM4-5, #100515 and #100510), CD8a (53-6.7, #100713), CD25 (PC61, #102008), IFNγ (XMG1.2, #505824), IL-4 (11B11, #504118) or IL-17A (TC11-18H10.1, #506904).
Validation	All the antibodies used in FACS are validated on the manufacturer's website for flow cytometry analyses of target proteins from mouse. The antibodies of PARP1, PARP2, PAR, β-catenin and β-actin are validated on the manufacturer's website for immunoblotting of target proteins from human and mouse. The antibodies of PARP1, PARP2, β-catenin and p65 are validated on the manufacturer's website for ChIP of target proteins from mouse.

Eukaryotic cell lines

Policy information about cell lines

Cell line source(s)	The luciferase-labeled bone-metastasis-prone MDA-MB-231 human breast cancer cell sub-line (MDA231-BoM-1833) (reference 12 in manuscript) was provided by J. Massague´. The luciferase-labeled Py8119 bone-metastatic-prone mouse mammary tumor cell line (reference 14 in manuscript) originally derived from spontaneous mammary tumors in C57BL/6 MMTV-PyMT female transgenic mice (reference 32 in manuscript) was provided by Dr. Lesley Ellies (UCSD). RAW264.7 macrophage cell line was from ATCC. The luciferase-labeled 4T1.2 mouse mammary tumor sub-line (referemce 13 in manuscript) was provided by Robin Anderson (Peter MacCallum Cancer Centre) and Yibing Kang (Princeton University).
Authentication	These cell lines were directly received from original and reliable sources, thus not further authenticated.
Mycoplasma contamination	The cell lines were tested negative for mycoplasma contamination.
Commonly misidentified lines (See ICLAC register)	No commonly misidentified cell line was used.

Animals and other organisms

Policy information about studies involving animals; ARRIVE guidelines recommended for reporting animal research

Laboratory animals	We used male or female of 6-8 week old PARP1 and PARP2 global knockout mice, PARP1 flox mice with or without LyzM-Cre, PARP2 flox mice with or without LyzM-Cre on C57BL/6 background. Female of 6-8 week old wild-type C57BL/6 mice (UTSW Breeding Core), BALB/cJ mice (UTSW Breeding Core) and athymic nude mice (Charles River, Strain# 553) were used. We used 2-7 month old female of MMTV-PyMT mice (Jackson laboratories, Stock # 022974) and MMTV-PyMT mice (Jackson laboratories, # 003553). Nude PARP1 and PARP2 global knockout mice were established by breeding PARP1 and PARP2 global knockout C57BL/6 mice with athymic nude mice, and 6-8 week old female were used.
Wild animals	This study did not involve wild animals.
Field-collected samples	This study did not involve samples collected from the field.
Ethics oversight	All protocols for mouse experiments were approved by the Institutional Animal Care and Use Committee of University of Texas Southwestern Medical Center.

Note that full information on the approval of the study protocol must also be provided in the manuscript.

Flow Cytometry

Plots

Confirm that:

- [x] The axis labels state the marker and fluorochrome used (e.g. CD4-FITC).
- [x] The axis scales are clearly visible. Include numbers along axes only for bottom left plot of group (a 'group' is an analysis of identical markers).
- [x] All plots are contour plots with outliers or pseudocolor plots.
- [x] A numerical value for number of cells or percentage (with statistics) is provided.

Methodology

Sample preparation	Bone marrow cells were isolated and filtered with a 100 µm cell strainer. Then cells were treated with ACK (Ammonium-Chloride-Potassium) lysis buffer (150 mM NH4Cl, 10 mM KHCO3, 1 mM EDTA, pH 7.2) for 3 min on ice. Cells were blocked with anti-CD16/32 (anti-Fcγ R III/II receptor, clone 93, 1:1000 dilution) for 20 min and then stained for 20 min with 7-AAD and antibodies (1:200 dilution) against CD45 (clone 30-F11, BD Biosciences), CD11b (clone M1/70), F4/80 (clone BM8), CD11c (clone N418), MHC II (clone M5/114.15.2), Gr1 (clone RB6-8C5), B220 (clone RA3-6B2), NK1.1 (clone PK136), CD3 (clone 17A2), CD4 (clone RM4-5), CD8a (clone 53-6.7) and CD25 (clone PC61). Regulatory T cells were analyzed by further treatment with Foxp3/ Transcription Factor Staining Buffer (Invitrogen) and stained with anti-FoxP3 (1:200 dilution, clone FJK-16s, Invitrogen). To analyze Type 1 and 2 T helper cells and T-helper 17 cells, bone marrow cells were stimulated with Cell Activation Cocktail plus Brefeldin A (Biolegend) for 4-6 hours after ACK treatment. After cell surface staining, cells were further treated with Intracellular Fixation & Permeabilization Buffer (Invitrogen) and stained with antibodies (1:200 dilution) against IFNγ (clone XMG1.2), IL-4 (clone 11B11) or IL-17A (clone TC11-18H10.1).
Instrument	Samples were analyzed on LSR II flow cytometer (BD Biosciences).
Software	Data was collected with the BD FACSDiva software and analyzed with the BD FlowJo V10 software.
Cell population abundance	Sorting was not performed.

Gating strategy

Using the FSC/SSC gating, debris was removed by excluding very low FSC and SSC, as well as low FSC and high SSC. The boundaries between unstained control cells and stained cells with each single antibody were used to distinguish between negative staining and specific positive antibody staining. The live cells were identified as 7-AAD negative cells. Then immune cells were identified as CD45+ cells. Among immune cells, immature myeloid cells were identified as CD11b+, Gr1+ cells; macrophages were identified as CD11b+, F4/80+ cells; dendritic cells were identified as CD11c+, MHCII+ cells; B cells were identified as B220+ cells; natural killer cells were identified as NK1.1+ cells; T cells were CD3+; T helper cells were CD3+, CD4+; T cytotoxic cells were CD3+, CD8a+; Treg cells were CD4+ CD25+ FoxP3+; Th1 cells were CD4+ IFNγ+; Th2 cells were CD4+ IL4+ and Th17 cells were CD4 + IL17A+. An example for the gating strategy is presented in Supplementary Fig. 11.

☒ Tick this box to confirm that a figure exemplifying the gating strategy is provided in the Supplementary Information.
